# Foreign body removal using flexible bronchoscopy in terminal cancer: A case report

**DOI:** 10.1097/MD.0000000000027620

**Published:** 2021-10-29

**Authors:** So-Yeon Jeon, Yeong-Hun Choe, Eun-Kee Song, Chang-Yeol Yim, Na-Ri Lee

**Affiliations:** a Division of Hematology and Oncology, Department of Internal Medicine, Jeonbuk National University Hospital, Jeonbuk National University Medical School, Jeonju, Republic of Korea; b Research Institute of Clinical Medicine, Jeonbuk National University, Biomedical Research Institute of Jeonbuk National University Hospital, Jeonju, Republic of Korea; c Division of Respiratory medicine and Allergy, Department of Internal Medicine, Jeonbuk National University Hospital, Jeonbuk National University Medical School, Jeonju, Republic of Korea.

**Keywords:** bronchoscopy, cancer, case report, foreign body

## Abstract

**Rationale:**

Pulmonary foreign body aspiration is a serious medical problem. The risk of foreign body aspiration into the airways increases considerably in patients with end stage cancer with reduced consciousness and impaired airway reflexes. However, few studies have reported on foreign body aspiration in the airways in patients with terminal cancer or receiving end-of-life care. Herein, we report the use of flexible bronchoscopy in patients with end-of-life cancer with pulmonary aspiration.

**Patient concerns:**

A 71-year-old man with neuroendocrine carcinoma was admitted to a palliative care unit for end-of-life care. He accidentally aspirated implant teeth into the airway with decreased consciousness and death rattle.

**Diagnosis:**

On chest x-ray, the foreign material was observed in the left main bronchus.

**Interventions:**

Despite concerns regarding the use of bronchoscopy given the deterioration of the overall organ function, flexible bronchoscopy was performed.

**Outcomes:**

Eventually, the foreign body was removed using a basket in the nasal cavity without major complications. The patient died comfortably after 7 days.

**Lessons:**

The possibility of patients in the palliative care unit with reduced consciousness and death rattle to aspirate foreign bodies into the airways must be carefully considered. Flexible bronchoscopy should be considered to carefully remove aspirated foreign bodies in the airway without any side effects, even in patients with terminal cancer or receiving end-of-life care.

## 1 Introduction

Foreign body aspiration into the airway can be life-threatening. Retrospective studies have suggested that the leading causes of tracheobronchial foreign body aspiration in adults are impaired airway reflexes associated with neurological disease, altered mental status due to alcohol or sedative use, dental procedures, and trauma with a decreased level of consciousness.^[1,2]^ Such situations are associated with decreased mental status, dysphagia, and/or impaired cough reflex.^[1,2]^ In dying patients, consciousness gradually decreases and the time spent asleep increases. Death rattles frequently occur in dying patients. The variation in the reported prevalence rates of death rattles is wide, 12% to 92%.^[3,4]^ The death rattle is caused by an accumulation of secretions in the pharynx and airways, and an absence of effective swallowing and coughing reflexes.^[4,5]^ During this time, the risk of aspiration increases when foreign substances enter the mouth. Information on tracheobronchial foreign body aspiration in patients with terminal cancer is limited.

Herein, we report the successful removal of an aspirated dental implant using flexible bronchoscopy in a patient with dying neuroendocrine carcinoma.

## 2 Case report

A 71-year-old man was referred to a palliative care unit for supportive care due to decreased cognitive function, abdominal pain in the right upper quadrant, and dyspepsia.

He had been diagnosed with neuroendocrine carcinoma of the stomach with multiple liver metastases seven months earlier. He received palliative chemotherapy comprising cisplatin and etoposide every 3 weeks. After 6 cycles, the liver mass had increased, and the chemotherapy was discontinued.

He was administered 12 μg/h of transdermal fentanyl patch for pain control, and received total parenteral nutrition due to the inability to ingest food. Seven days after the admission, the patient was unable to spontaneously expectorate sputum due to a decreased level of consciousness, and hyoscine butylbromide was administered to control the death rattle. After 3 days, the patient's consciousness decreased to semi-coma with a worsened death rattle, and the dosage of hyoscine butylbromide was increased to 100 mg/day. However, his death rattle was not well-controlled, and the sputum had to be suctioned manually. Two days after suctioning, the patient's central incisor implant had accidentally dislodged during suctioning and was aspirated.

**Figure 1:**
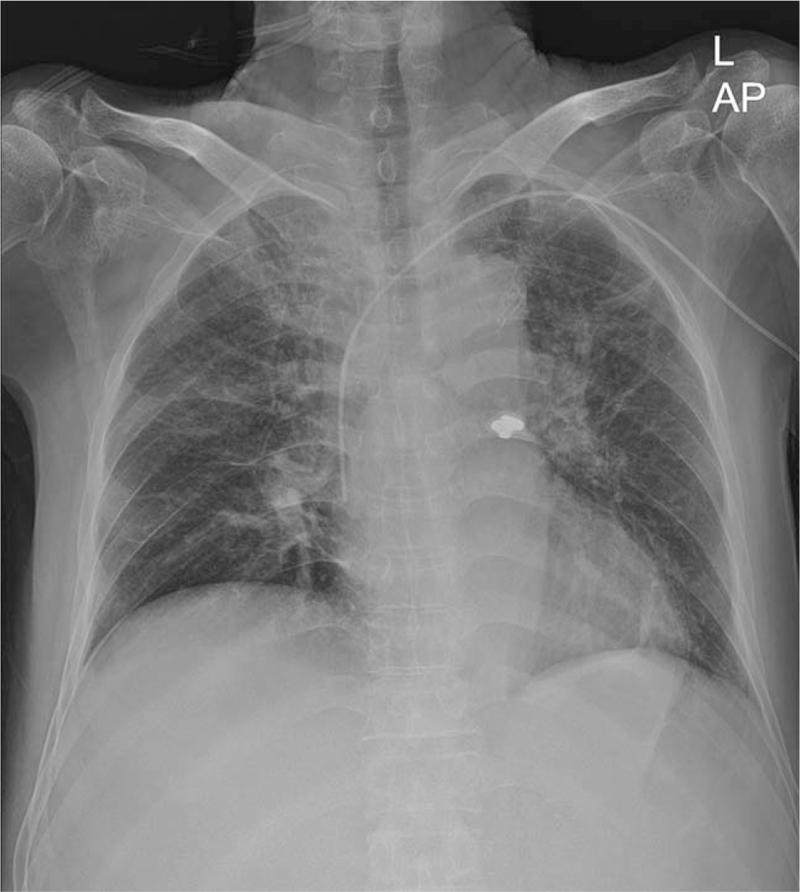
Chest x-ray showing a radio-opaque foreign material in the left main bronchial region.

The vital signs showed an elevated pulse rate of 125 beats per minute, breathing rate of 28 breaths per minute, and temperature of 38.7°C. Oxygen saturation was measured using pulse oximetry and ranged from 90% to 92%. We managed with supplemental oxygen and antibiotics and piperacillin/tazobactam was added. On physical examination, breathing sounds were decreased, and wheezing sounds were noted in the left lung field. On chest radiography, the foreign material was observed in the left bronchus (Fig. 1).

The patient's family did not want him to die with a foreign body in his trachea. Based on the patient's symptoms and the family's and pulmonologist's opinions, bronchoscopy was considered.

**Figure 2:**
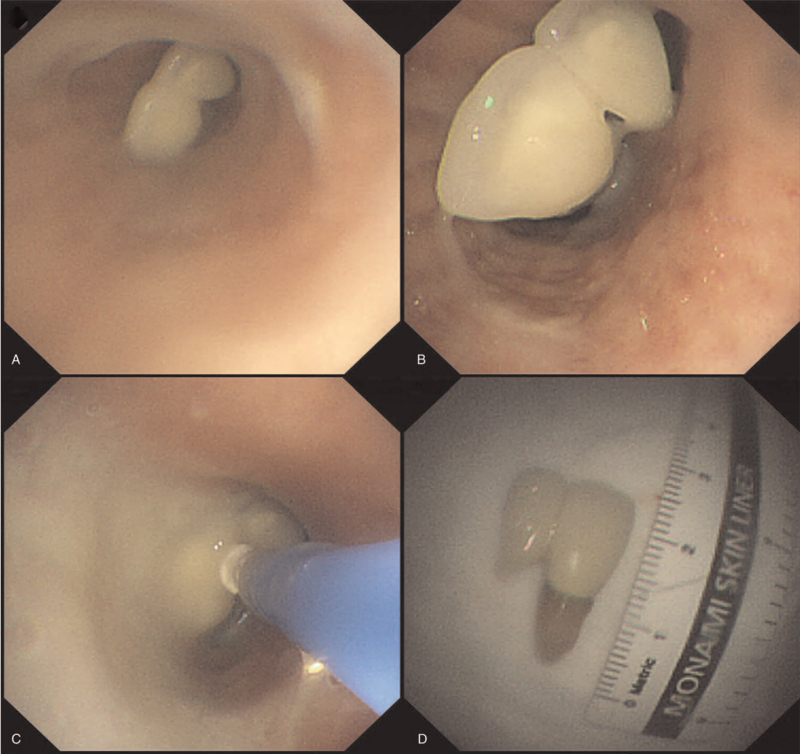
Flexible bronchoscopy image. (A) Both sides of the bronchi are full of sputum, and (B) a foreign body in the left main bronchus. (C) Foreign body retrieved by attaching to the cryoprobe and large amount of aspirated sputum. (D) Foreign body: A 2-cm dental implant.

Therefore, flexible bronchoscopy (BF-1TQ290, Olympus, Tokyo, Japan) was performed. Sedation was not performed because the patient was unconscious. Oxygen saturation was 88% to 94% on supplemental oxygen via a nasal cannula at 10 L/min during the procedure. The bronchoscopic findings, saliva, and sputum aspiration were continued, and both sides of the bronchi were observed to be full of sputum (Fig. 2A). A foreign body was observed in the left main bronchus (Fig. 2B) and was retrieved to the nasal cavity using a cryoprobe. However, the patient was unable to cooperate with the removal using the cryoprobe in the nasal cavity and re-aspirated the implant. Sputum was constantly sucked into the airway (Fig. 2C). Eventually, the foreign body was removed using a basket in the nasal cavity, without complications. The foreign body was a 2-cm dental implant (Fig. 2D).

After the procedure, the patient had decreased sputum production, and his breathing became less labored. His blood pressure gradually decreased, and he died uneventfully 7 days later.

## 3 Discussion

Tracheobronchial foreign body aspiration is a serious medical problem associated with high morbidity and mortality rates. In adults, the incidence rate increases after 60 years of age when the airway protection mechanism begins to function abnormally.^[2]^ The prevalence of cerebrovascular and degenerative neurological diseases increases with aging.^[6]^ Medications such as anticholinergics, antipsychotics, or anxiolytics, which can cause impairment of the cough reflex and/or swallowing, are often prescribed.^[7]^ These drugs are widely used in the hospice palliative care field, and may be one of the reasons for tracheobronchial foreign body aspiration in patients with terminal cancer.^[8,9]^ In this patient, anticholinergics, hyoscine butylbromide were also administered to reduce death rattle.

The death rattle is the noise produced by the oscillatory movements of secretions in the upper airways in association with the inspiratory and expiratory phases of respiration.^[9]^ Death rattle is strongly associated with the occurrence of death within 2 to 3 days in patients with cancer^[10,11]^ and more frequently occurs when the dying phase is prolonged.^[3,12]^ It is caused by an accumulation of secretions in the airways because of the absence of effective reflexes during coughing and swallowing. Therefore, the risk of bronchial foreign body aspiration is high during death rattle in a patient terminally ill of cancer. However, information on bronchial foreign body aspiration in such patients and those receiving end-of-life care is insufficient.

Therefore, there are limitations on bronchial foreign body aspiration that can only be compared with adult data. Bronchial foreign bodies were more commonly located in the right bronchus, with approximately 60% in the right bronchus and 40% in the left bronchus in adult patients.^[13,14]^ Furthermore, the most common symptoms were sudden onset of choking, intractable cough, fever, breathlessness, and wheezing.

In adults, substances that are be aspirated are diverse. According to the report by Ng et al, among the 111 adult patients with foreign body aspiration, 74 (66%) had aspirated food material (42 bones [38%], 24 food [21%], 8 pills [7%]), teeth, artificial teeth, and dental instruments were aspirated in 15% of 16 patients.^[14]^ In another study in adults, metallic foreign bodies were the most common in 16 patients (41%) among 39 patients who knew the foreign body type, followed by organic in 10 patients (25.6%), and tooth in 8 patients (20.5%).^[15]^ In 17 geriatric patients, 7 (41%) had aspirated chicken and fish bone, 7 (41%) had aspirated plants and melon seeds, dentures, and 3 (18%) had aspirated tracheal cannulas.^[13]^ Food material was most commonly aspirated in adults, and teeth, artificial teeth, and dental instruments were aspirated in approximately 15% to 20% of cases.

Airway support is the initial management of bronchial foreign body aspiration. Once the airway is secured, appropriate imaging may assist in localizing the foreign body. Conventional chest radiography is the imaging modality of choice in suspected cases of bronchial foreign body aspiration. Radiopaque materials, such as metals or teeth, can be easily identified on chest radiography. However, organic materials, such as food, are the most frequently aspirated foreign materials and are not radiopaque.^[2,16]^ Such materials are less likely to be detected on chest x-rays. In this patient, the implanted teeth were aspirated; hence, chest radiography was able to determine the location of the lesion accurately.

Both rigid and flexible bronchoscopies have been utilized in the removal of foreign bodies.

Flexible bronchoscopy has been reported as the most effective diagnostic and treatment method for airway foreign body removal because of its ease of use and lack of requirement for general anesthesia.^[13]^ The success rate for flexible bronchoscopy, as reported by a recent systematic review, was 61% to 100%.^[15]^ Failure of flexible bronchoscopy was observed in case of a longer duration between aspiration and bronchoscopic removal and a more peripheral airway foreign body. As bronchoscopy became more readily available, the death rate from aspiration decreased from 50% to <1%.^[13]^

In this patient, the aspiration of teeth and dentures in a decreased level of consciousness mainly occurred during endotracheal intubation and extubation; the incidence of perianesthesia dental injury was 0.04% to 12.08%.^[17]^ After intubation in a patient with sudden loss of consciousness due to intracerebral hemorrhage, 2 teeth were aspirated into each bronchus. Both teeth were removed using rigid bronchoscopy, and the patient was confirmed to have multiple loose teeth on dental examination. As loose artificial teeth were aspirated in our patient, it is important to check for loose teeth in patients with reduced consciousness.^[17]^ Cases have been reported of patients with Parkinson's disease and patients recovering from subarachnoid hemorrhage, and decreased functional chewing units and decreased sensation of palate and cough reflex were also the main causes of foreign body aspiration.^[18,19]^

Irrespective of the treatment success rate, the use of bronchoscopy in patients with terminal cancer has some concerns due to the deterioration of overall organ function.

Even in our patient, concerns regarding the risk of bronchoscopy in an unconscious patient at the end of the life process. However, due to the patient's fever and dyspnea, the caregiver desired to remove the foreign body, and flexible bronchoscopy was performed as a necessary procedure to alleviate the patient's suffering. Furthermore, it was possible to remove the foreign body with the rapid judgment and procedure of a skilled pulmonologist with extensive experience in bronchoscopy.

However, owing to the lack of patient cooperation during bronchoscopy, the foreign body was difficult to retrieve to the nasal cavity. Fortunately, the foreign body was removed using a basket without any major complications, and the aspirated sputum was also removed such that the patient's breathing could be more stable.

When the patient entered the final phase of terminal cancer with a death rattle, the foreign body was aspirated into the bronchus. To our knowledge, this report is the first to report the safe removal of an aspirated foreign body using flexible bronchoscopy.

Patients with terminal cancer with reduced consciousness and death rattle must be carefully observed for the risk of aspirating foreign bodies, especially loose teeth, into the bronchus.

Although this article suggests that the removal of foreign substances through flexible bronchoscopy is relatively safe, even in patients with cancer in the end-of-life period, flexible bronchoscopy should be carefully performed for foreign bodies in the airway.

## Author contributions

**Conceptualization:** So-Yeon Jeon, Eun-Kee Song.

**Investigation:** Yeong-Hun Choe, Na-Ri Lee.

**Supervision:** Chang-Yeol Yim.

**Writing – original draft:** Na-Ri Lee.

**Writing – review & editing:** So-Yeon Jeon.

## References

[1] Limper AH Prakash UB. Tracheobronchial foreign bodies in adults. Ann Intern Med 1990;112:604–9. 2327678 10.7326/0003-4819-112-8-604

[2] Boyd M Chatterjee A Chiles C Chin R Jr. Tracheobronchial foreign body aspiration in adults. South Med J 2009;102:171–4. 19139679 10.1097/SMJ.0b013e318193c9c8

[3] Bennett M Lucas V Brennan M. Using anti-muscarinic drugs in the management of death rattle: evidence-based guidelines for palliative care. Palliat Med 2002;16:369–74. 12380654 10.1191/0269216302pm584oa

[4] Mercadamte S. Death rattle: critical review and research agenda. Support Care Cancer 2014;22:571–5. 24253734 10.1007/s00520-013-2047-5

[5] Bennett M Lucas V Brennan M Hughes A O’Donnell V Wee B. Using anti-muscarinic drugs in the management of death rattle: evidence-based guidelines for palliative care. Palliat Med 2002;16:369–74. 12380654 10.1191/0269216302pm584oa

[6] Marik PE Kaplan D. Aspiration pneumonia and dysphagia in the elderly. Chest 2003;124:328–36. 12853541 10.1378/chest.124.1.328

[7] Vergis EN Brennen C Wagener M Muder RR. Pneumonia in long-term care: a prospective case-control study of risk factors and impact on survival. Arch Intern Med 2001;161:2378–81. 11606155 10.1001/archinte.161.19.2378

[8] Wildiers H Dhaenekint C Demeulenaere P. Atropine, hyoscine butylbromide, or scopolamine are equally effective for the treatment of death rattle in terminal care. J Pain Symptom Manage 2009;38:124–33. 19361952 10.1016/j.jpainsymman.2008.07.007

[9] Mercadante S Marinangeli F Masedu F. Hyoscine butylbromide for the management of death rattle: sooner rather than later. J Pain Symptom Manage 2018;56:902–7. 30172864 10.1016/j.jpainsymman.2018.08.018

[10] Wildiers H Menten J. Death rattle: prevalence, prevention and treatment. J Pain Symptom Manage 2002;23:310–7. 11997200 10.1016/s0885-3924(01)00421-3

[11] Hui D dos Santos R Chisholm G. Clinical signs of impending death in cancer patients. Oncologist 2014;19:681–7. 24760709 10.1634/theoncologist.2013-0457 PMC4041673

[12] Kåss RM Ellershaw J. Respiratory tract secretions in the dying patient: a retrospective study. J Pain Symptom Manage 2003;26:897–902. 14527758 10.1016/s0885-3924(03)00292-6

[13] Lin L Lv L Wang Y Zha X Tang F Liu X. The clinical features of foreign body aspiration into the lower airway in geriatric patients. Clin Interv Aging 2014;9:1613–8. 25284994 10.2147/CIA.S70924 PMC4181443

[14] Ng J Kim S Chang B. Clinical features and treatment outcomes of airway foreign body aspiration in adults. J Thorac Dis 2019;11:1056–64. 31019795 10.21037/jtd.2018.12.130 PMC6462713

[15] Sehgal IS Dhooria S Ram B. Foreign body inhalation in the adult population: experience of 25,998 bronchoscopies and systematic review of the literature. Respir Care 2015;60:1438–48. 25969517 10.4187/respcare.03976

[16] Baharloo F Veyckemans F Francis C Biettlot MP Rodenstein DO. Tracheobronchial foreign bodies: presentation and management in children and adults. Chest 1999;115:1357–62. 10334153 10.1378/chest.115.5.1357

[17] Jiang XJ Zhang WY. Successful experience in dealing with tooth aspiration after extubation: a case report. Ann Palliat Med 2021;10:8420–4. 33894707 10.21037/apm-20-2541

[18] Chen K Ye M Shen X. Anesthetic management for retrieval of a large aspirated denture in a patient with Parkinson's disease. J Clin Anesth 2017;43:59–60. 28985585 10.1016/j.jclinane.2017.09.016

[19] Jansen J van der Maarel-Wierink CD Dubois L. [Acute respiratory distress in a frail older patient: spontaneous tooth aspiration]. Ned Tijdschr Tandheelkd 2020;127:282–5. 32609098 10.5177/ntvt.2020.05.19132

